# Mesenchymal stem/stromal cells enhance engraftment, vasculogenic and pro-angiogenic activities of endothelial colony forming cells in immunocompetent hosts

**DOI:** 10.1038/s41598-017-13971-3

**Published:** 2017-10-19

**Authors:** Abbas Shafiee, Jatin Patel, James S. Lee, Dietmar W. Hutmacher, Nicholas M. Fisk, Kiarash Khosrotehrani

**Affiliations:** 1 The University of Queensland, UQ Centre for Clinical Research, Brisbane, 4029 QLD Australia; 2 Queensland University of Technology, Brisbane, 4000 QLD Australia; 3 Centre for Advanced Prenatal Care, Royal Brisbane & Women’s Hospital, Brisbane, 4029 QLD Australia; 4 The University of Queensland, UQ Diamantina Institute, Brisbane, 4102 QLD Australia

## Abstract

The clinical use of endothelial colony forming cells (ECFC) is hampered by their restricted engraftment. We aimed to assess engraftment, vasculogenic and pro-angiogenic activities of ECFC in immunocompetent (C57BL/6: WT) or immunodeficient (*rag1* ^*−/−*^C57BL/6: Rag1) mice. In addition, the impact of host immune system was investigated where ECFC were co-implanted with mesenchymal stem/stromal cells (MSC) from adult bone marrow (AdBM-MSC), fetal bone marrow (fBM-MSC), fetal placental (fPL-MSC), or maternal placental (MPL-MSC). Transplantation of ECFCs in Matrigel plugs resulted in less cell engraftment in WT mice compared to Rag1 mice. Co-implantation with different MSCs resulted in a significant increase in cell engraftment up to 9 fold in WT mice reaching levels of engraftment observed when using ECFCs alone in Rag1 mice but well below levels of engraftment with MSC-ECFC combination in Rag1 recipients. Furthermore, MSCs did not reduce murine splenic T cell proliferation in response to ECFCs *in vitro*. ECFCs enhanced the murine neo-vascularization through paracrine effect, but with no difference between Rag1 and WT mice. In conclusions, the host adaptive immune system affects the engraftment of ECFCs. MSC co-implantation improves ECFC engraftment and function even in immunocompetent hosts mostly through non-immune mechanisms.

## Introduction

Cardiovascular diseases (CVD) are the leading cause of morbidity and death in the developed world^1,2^. Restoring blood supply to the ischemic tissues is suggested to be an integral and promising approach in their treatment. In 1997, Asahara *et al*. described a new population of cells, endothelial progenitors, with potential to home to ischemic tissues and contribute in neovascularization^3^. Since their initial description, their potential therapeutic use has attracted much interest, particularly for CVD. Although bone marrow (BM) was initially considered as the main source of these progenitors^4^, more stringent definitions through exclusion of hematopoietic cells^5,6^, and restriction of their lineage to the endothelium allowed progenitor isolation from other sources including perinatal tissues^7^. In particular, *ex vivo* culture of late outgrowth endothelial progenitors from human cord blood (CB) and more recently from the human term placenta^8,9^ has further strengthened this progenitor definition by establishing a self-renewal hierarchy among endothelial cells and naming these progenitors as endothelial colony-forming cells (ECFCs)^10–13^. The *in vivo* vasculogenic potential^14–16^ of ECFCs raises considerable prospect for their use in human clinical trials. Of significance, placental ECFCs, available in large quantities from a typically discarded tissue, reinforce the possibility of large scale ECFCs isolation for clinical applications^17^. This implies that ECFCs can be used as an off the shelf product in allogeneic transplantation. However, neo-vascularization of ischemic tissues using ECFC has been hampered by their restricted cell reconstitution and engraftment upon transplantation^18^. We and others have demonstrated that unlike mesenchymal stem/stromal cells (MSCs), ECFCs are not able to attenuate allogeneic immune responses^9,19–21^. This characteristic could partially explain the low engraftment observed after transplantation.

We and other groups have shown that ECFCs neovascularization could be improved through co-transplantation with MSCs^14,22–24^. When the transplanted cells were not confronted with an allogeneic immune system, the advantageous effects of MSCs were explained to be due to their role as supportive cells in stabilizing the newly-formed vasculature mainly through Notch signalling^14,22^. However, in the presence of alloreactive immune cells, this positive outcome upon co-engraftment could be further explained by MSCs immunomodulatory effect^23^. It has been well documented that MSCs are immunosuppressive and able to modulate immune responses against third party antigens or cells (reviewed in^19–21^) including immune responses against ECFCs^23^. However, the advantages of MSCs functions on levels of ECFC engraftment has not been addressed before and most studies to date were conducted in immunodeficient animal models or using autologous EPCs^25,26^.

Here, we hypothesized that the host adaptive immune system can impact on ECFCs engraftment levels and this effect can be modulated through MSC co-transplantation. Therefore, to assess the importance of MSCs’ effects on ECFCs function, we evaluated ECFCs engraftment levels upon co-transplantation with MSCs in immunocompetent (WT) and immunodeficient (*rag1* ^*−/−*^: Rag1) mice from the same genetic background (C57Bl/6). Our results demonstrate ECFC engraftment levels are modulated by the host adaptive immune system, and can be improved in immunocompetent mice by co-injecting ECFCs with MSCs.

## Results

### Immunodeficient mice support higher levels of ECFC engraftment

**Figure 1:**
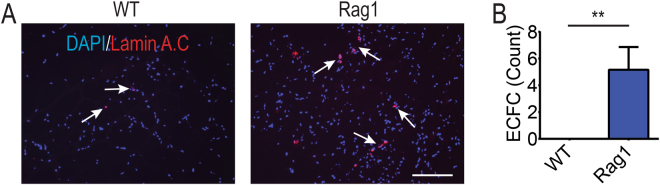
Negligible endothelial colony forming cells (ECFCs) could survive in immunocompetent (C57BL/6: WT) and immunocompromised *rag1* ^*−/−*^ (Rag1) mice. (**A**) 5 × 10^5^ cells were mixed with Matrigel and subcutaneously injected into mice, and the number of engrafted human cells counted by human nuclear Lamin A/C staining (red) at day 7. Arrows indicate human cells, scale bar: 100 µm. (**B**) Transplantation in Rag1 mice resulted in a significant increase in cell engraftment compared to WT mice (n = 5 mice) (**P < 0.01). Bars represent mean ± SD.

To evaluate the impact of the adaptive immune system on ECFC engraftment, 5 × 10^5^ ECFCs were injected into WT or Rag1 mice and the number of engrafted cells measured by human nuclear Lamin A/C staining. Only rare ECFC could survive in WT mice after 7 days upon careful consideration of multiple sections, whereas at the same time a few ECFC could be recovered in Rag1 mice in each section (Fig. 1A, n = 5 mice). Cell quantification demonstrated that transplantation of ECFC alone in Rag1 mice resulted in higher engraftment levels compared to WT (5.16 ± 1.6 cells per field of view vs 0 +/− 0.1, Figs 1B, **P < 0.01).

### MSC from different sources enhance ECFC engraftment

**Figure 2:**
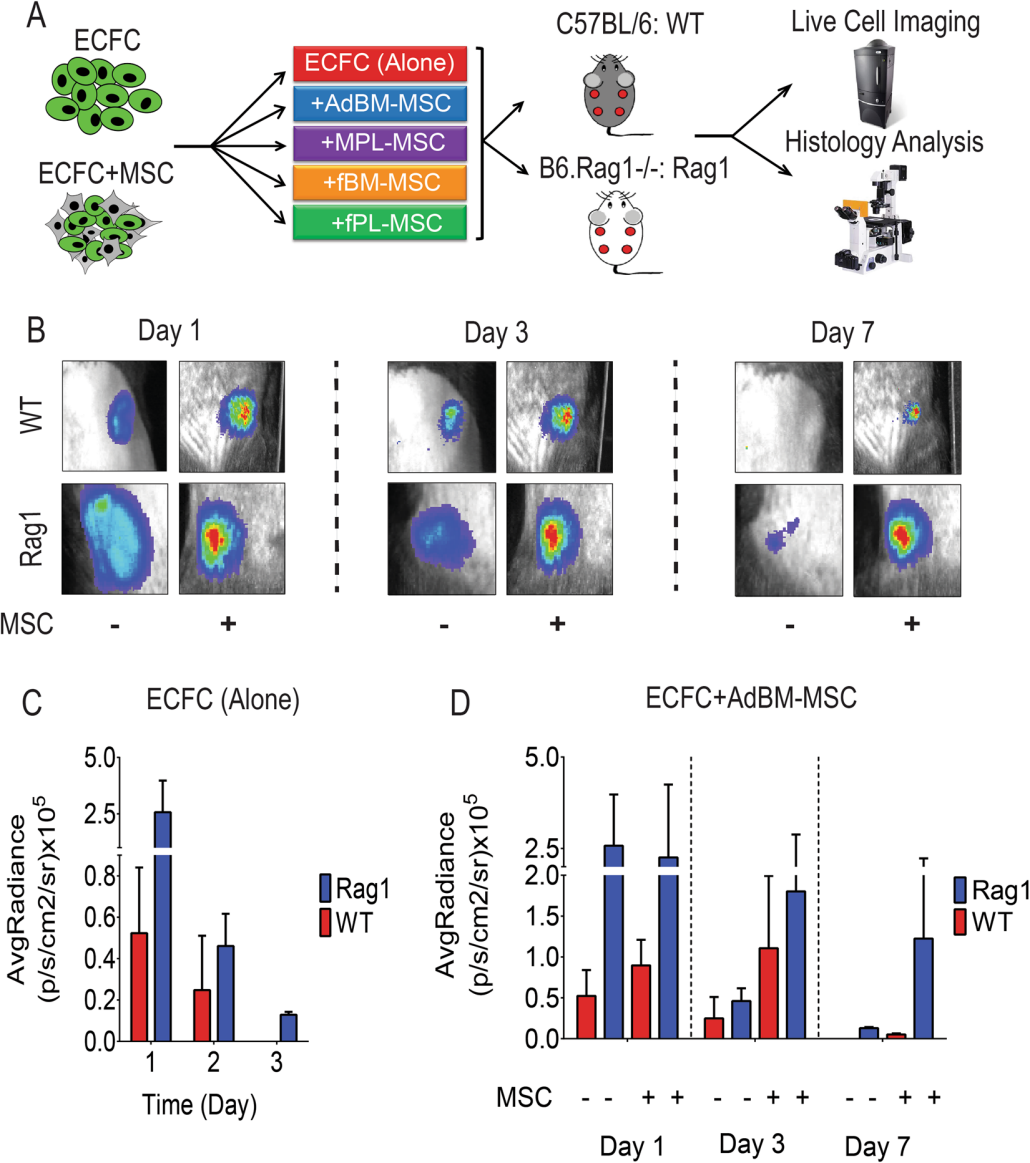
Co-transplantation with mesenchymal stem/stromal cells (MSC) results in increased endothelial colony forming cells (ECFC) survival. (**A**) GFP-tagged ECFCs were engrafted alone or with human MSC from adult bone marrow (AdBM-MSC), first trimester fetal bone marrow (fBM-MSC), term placenta fetal MSC (fPL-MSC) or maternal MSC (MPL-MSC) in immunocompetent (C57BL/6: WT) and immunocompromised *rag1* ^*−/−*^ (Rag1) mice. Live cell imaging was conducted on days 1, 3, and 7 post engraftment. (**B**) Representative bioluminescence images after ECFC alone or ECFC and AdBM-MSC co-transplantation in WT and Rag1 mice. (**C,D**) Time course monitoring of bioluminescence in WT and Rag1 mice. (**C**) ECFC alone (n = 3 mice), (**D**) ECFC alone versus ECFC and AdBM-MSC. Bars represent mean ± SD.

Given the immunosuppressive functions of MSCs, and their potential to enhance ECFC engraftment^24^, we next assessed the impact of MSC co-injection on ECFC engraftment. eGFP and luciferase-tagged ECFCs were co-implanted in Matrigel plugs in WT or Rag1 mice, either alone or associated with MSC from adult bone marrow (AdBM-MSC), fetal bone marrow (fBM-MSC), fetal placental (fPL-MSC), or maternal placental (MPL-MSC), and compared to ECFC alone (Fig. 2A).

*In vivo* bioluminescence imaging was performed on days 1, 3 and 7 post cell transplantation. There was a significant difference on day 1 in average radiance between WT and Rag1 mice (Fig. 2B Day1, and Fig. 2C, n = 3 mice). Time course monitoring revealed a sharp decrease in bioluminescence intensity from day 1 to 7 post-implantation of ECFCs alone (Fig. 2B Day3 and Day 7, and Fig. 1C) in both WT and Rag1 mice. ECFC related bioluminescence was higher at all time points in Rag1 animals and no bioluminescence could be detected in WT mice at D7 (Fig. 2B Day7, and Fig. 2C). When co-injected with MSCs from different sources, ECFC related bioluminescence was significantly increased in WT and Rag1 mice (Fig. 2B, representative images for AdBM-MSC, and Fig. 2D and Supp. Fig. 1A–C). Of note, BM-MSCs of fetal or adult origin were particularly efficient in maintaining ECFC engraftment over time. While WT mice showed a sharp decrease in bioluminescence intensity between days 1 to 7 (Fig. 2D), ECFC bioluminescence in Rag1 remained relatively stable (Fig. 2D).

**Figure 3:**
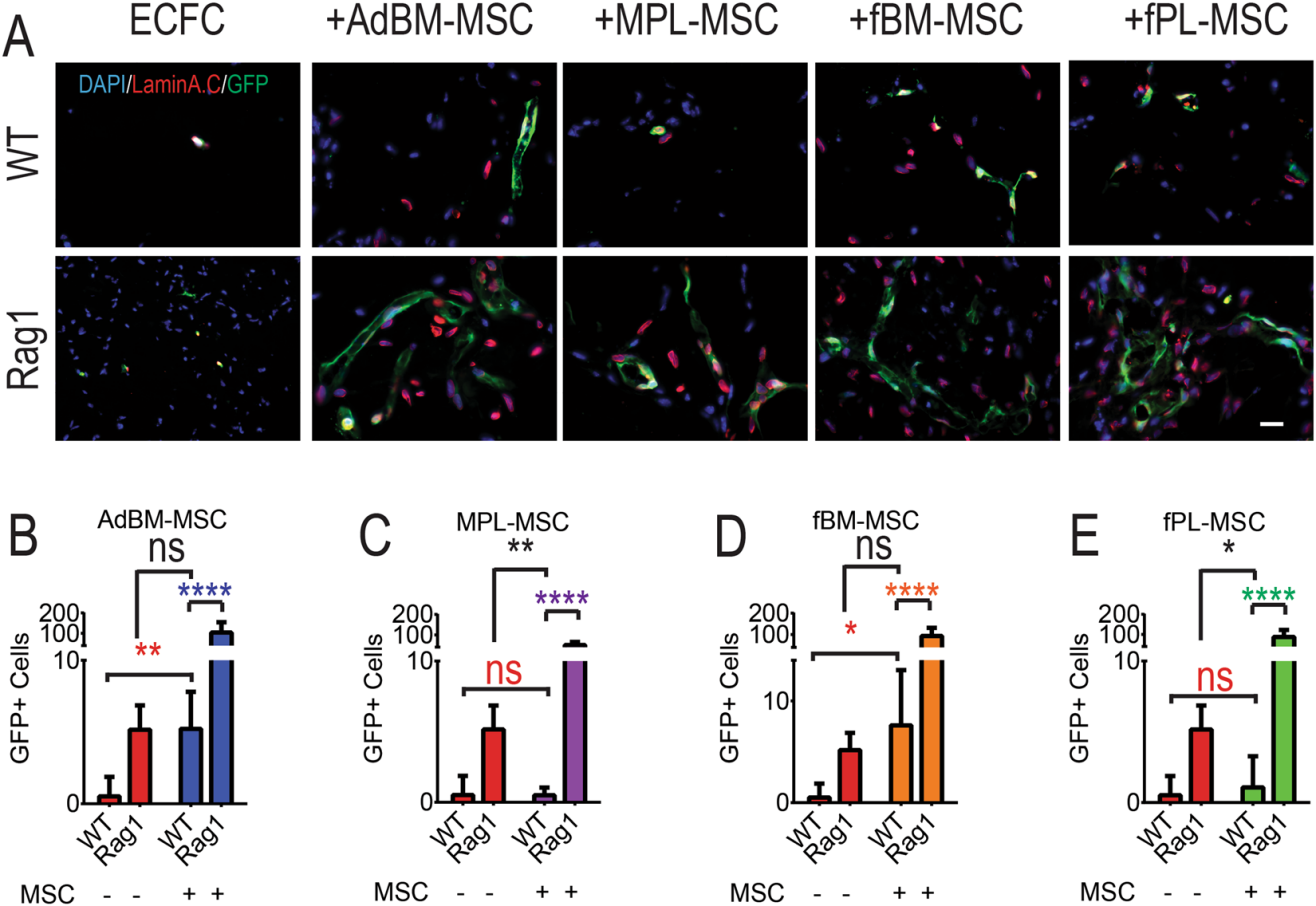
Mesenchymal stem/stromal cells (MSC) co-transplantation increases endothelial colony forming cells (ECFC) survival. (**A**) Co-staining of GFP+ cells (ECFCs) with human specific Lamin A.C antibody (red) in sections of Matrigel plugs injected in immunocompetent (WT) and immunocompromised (*rag1* ^*−/−*^: Rag1) mice at Day 7 (Scale bar: 20 µm). (**B–E**) Quantification of the number of GFP+ cells detected in sections stained as above (n = 5 mice). MSC and ECFC co-injection resulted in increased ECFC number (GFP+). (**B**) ECFC alone versus ECFC co-implantation with AdBM-MSC. (**C**) ECFC alone versus ECFC co-implantation with MPL-MSC. (**D**) ECFC alone versus ECFC co-implantation with fBM-MSC. (**E**) ECFC alone versus ECFC co-implantation with fPL-MSC. *P < 0.05, **P < 0.01, ***P < 0.005, ns: non-significant. AdBM-MSC: MSC from adult bone marrow, MPL-MSC: maternal term placental MSC, fBM-MSC: first trimester fetal bone marrow MSC, and fPL-MSC: fetal term placental MSC. Data presented as mean ± SD.

To confirm findings we next examined sections from plugs harvested 7 days post engraftment. Transplanted cells were identified using human specific nuclear marker Lamin A/C. In control experiments, in the absence of any transplanted cell, no GFP+ or Lamin A/C expression could be detected in Matrigel plugs (data not shown). While plugs which were embedded with ECFC alone contained few cell numbers, MSC-ECFC transplanted plugs displayed numerous eGFP-ECFC (Fig. 3A). Altogether, MSC co-implantation resulted in a remarkable increase in ECFC cell numbers at day 7 reaching 8–18 fold in Rag1 compared to 1–9 fold in WT mice (Fig. 3A–E). As demonstrated in Fig. 3B, co-engraftment with AdBM-MSC resulted in the highest ECFC engraftment levels in WT (8.1 fold, P < 0.01, Mann Whitney test, n = 5 mice). There was no significant difference in ECFCs engraftment levels between Rag1 animals transplanted with ECFCs alone and WT mice transplanted with combination of ECFC and AdBM-MSC (Fig. 3B, P = 0.76). In contrast MPL-MSC showed the weakest level of MSC support of ECFC engraftment in Rag1 mice (8.6 fold, ****P < 0.001) and displayed no contribution of MSCs to ECFC engraftment in WT mice (n = 5 mice, P = 0.75, Fig. 3C, Mann Whitney test). Similar to AdBM-MSC, there was no significant difference in ECFC engraftment levels between Rag1 animals transplanted with ECFC alone and WT mice transplanted with combination of ECFC and fBM-MSC (n = 5 animals. Fig. 3D, P = 0.6). Finally, ECFC engraftment was also supported in co-implantation with fPL-MSC in Rag1 (15.0 fold, P < 0.005) but not in WT mice (n = 5 mice, 1.9 fold, P = 0.4, Fig. 3E).

To determine whether MSCs were affecting T cell responses, C57Bl/6 mouse splenocytes were labelled with CFSE and then cultured for 48 h either alone or with ECFC only or ECFC + MSC combined (Supp. Fig. 2A). After incubation CD3+ cells were gated and the dilution of CFSE measured (Supp. Fig. 2B). The CFSE labelling assay indicated an increase in T cell proliferation in cultures containing human cells as compared to controls containing only murine splenocytes (Supp. Fig. 2B,C). Importantly, incubation with MSC + ECFC did not reduce T cell proliferation compared to incubation with ECFC alone (Supp. Fig. 2C, n = 4).

### Co-injection with MSCs increases formation of human vessel-like structures

**Figure 4:**
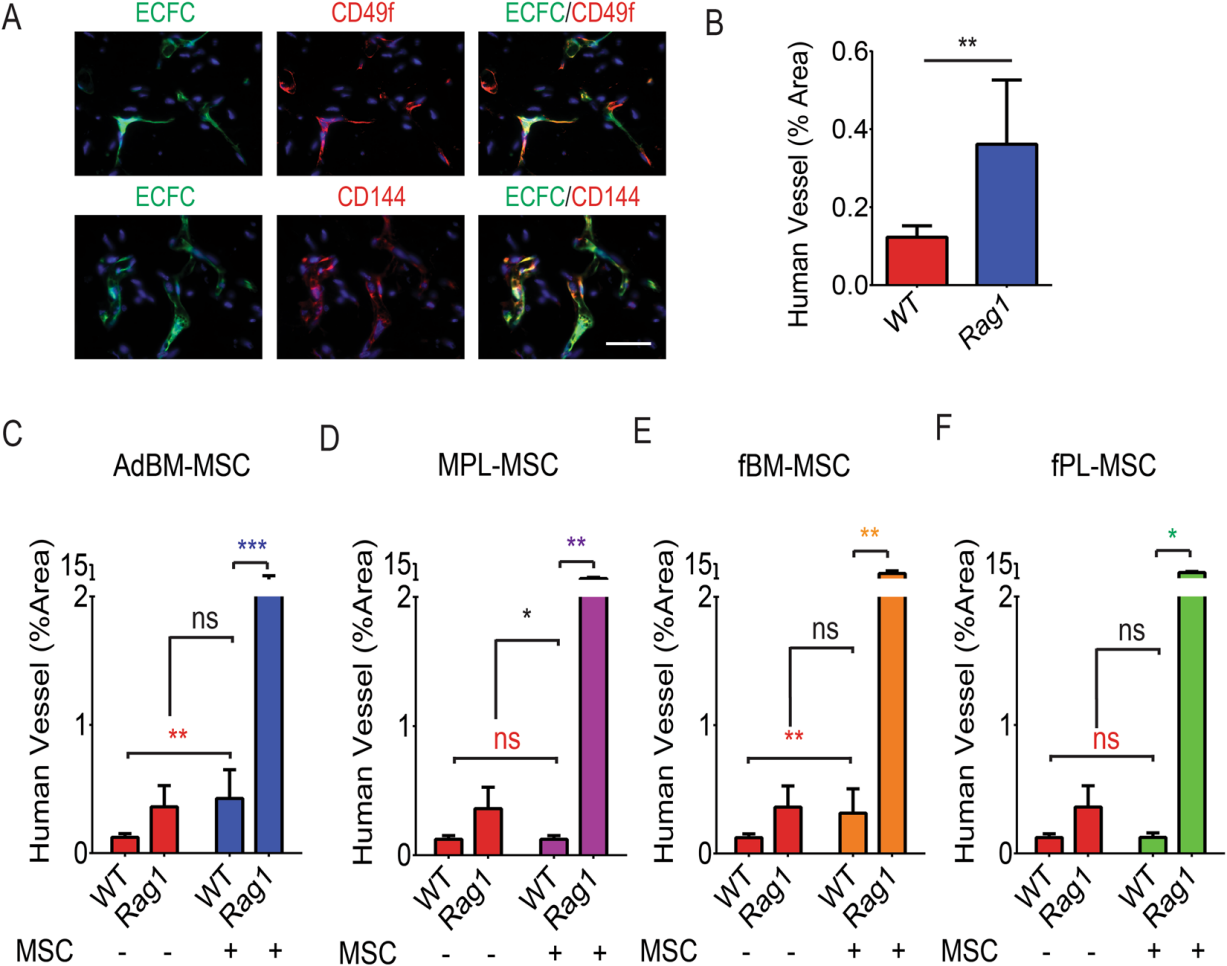
Co-implantation results in increased human vessel area at the transplantation site. (**A,B**) To assess the formation of human vessels, slides were co-stained with human CD49f and CD144 antibodies and scanned for GFP+ tube structures. The surface represented by GFP+ structures was measured as a percentage of the surface of the Matrigel plug on the section. (**A**) Representative images for AdBM-MSC + ECFC implantation in immunocompromised (*rag1* ^*−/−*^: Rag1) mice. Scale bar: 20 µm. (**B**) Comparison of plugs containing ECFC alone in immunocompetent (WT) and Rag1 mice. (**C**) ECFC alone versus ECFC co-implantation with AdBM-MSC. (**D**) ECFC alone versus ECFC co-implantation with MPL-MSC. (**E**) ECFC alone versus ECFC co-implantation with fBM-MSC. (**F**) ECFC alone versus ECFC co-implantation with fPL-MSC. *P < 0.05, **P < 0.01, ***P < 0.005, ns: non-significant. AdBM-MSC: MSC from adult bone marrow, MPL-MSC: maternal term placental MSC, fBM-MSC: first trimester fetal bone marrow MSC, and fPL-MSC: fetal term placental MSC. (n ≥ 4 mice). Data presented as mean ± SD.

GFP+ structures after ECFC implantation alone were minimal regardless of immune status. The human origin and vascular nature of these structures was confirmed by co-staining with anti-human CD49f and CD144 antibodies (Fig. 4A, representative image for AdBM-MSC + ECFC). Quantification revealed that transplantation of ECFC alone in Rag1 mice resulted in 3 fold increase in human vessel-like structures (CD144 positive area) compared to WT mice (n ≥ 4 mice. Fig. 4B, **P < 0.01). Upon co-implantation, AdBM-MSC strongly supported ECFC derived vessel-like structure formation in the implantation site in WT mice, narrowing the difference with Rag1 mice (3.5 fold, Fig. 4C). The level of human vessel-like structures in Rag1 mice transplanted with ECFCs alone compared to WT animals transplanted with combination of ECFCs and AdBM-MSC was similar (Fig. 4C, P = 0.6). However MPL-MSC co-injection showed no difference in human vessel area compared to ECFC injection alone in WT mice (n ≥ 4 mice for each group. Fig. 4D). Additionally, fBM-MSCs supported ECFC derived capillary-like formation in the implantation site in WT mice (n = 5 mice, 2.6 fold, Fig. 4E) reaching levels observed in Rag1 mice transplanted with ECFCs alone (Fig. 4E, P = 0.8). Finally, fPL-MSC co-injection showed no difference in human vessel area compared to ECFC injection alone in WT mice (n = 5 mice, Fig. 4F).

Of interest, all MSC subtypes increased the area of ECFC derived capillary-like structures in Rag1 mice compared to WT (Fig. 4C–F, P < 0.05). Finally, none of the MSC populations delivered alone could form new human vessels (data not shown) as previously reported^27,28^ and suggesting their function as supportive cells.

### The paracrine activity of ECFCs is independent of the immune status of the host and can be improved by MSC co-implantation

It has been proposed that ECFCs as well as MSCs improve angiogenesis through paracrine function and do not necessarily act through engraftment and differentiation^14,15^. To assess the influence of the host immune system on the paracrine activity of human stem/progenitor cells, we evaluated mouse vessel density in Matrigel implants at D7 using a mouse specific anti-CD31 antibody. In control, experiments without cells delivered to the implant, no major vascularization could be found, with no difference by immune status (n = 5 mice, P > 0.9, Supp. Fig. 3).

**Figure 5:**
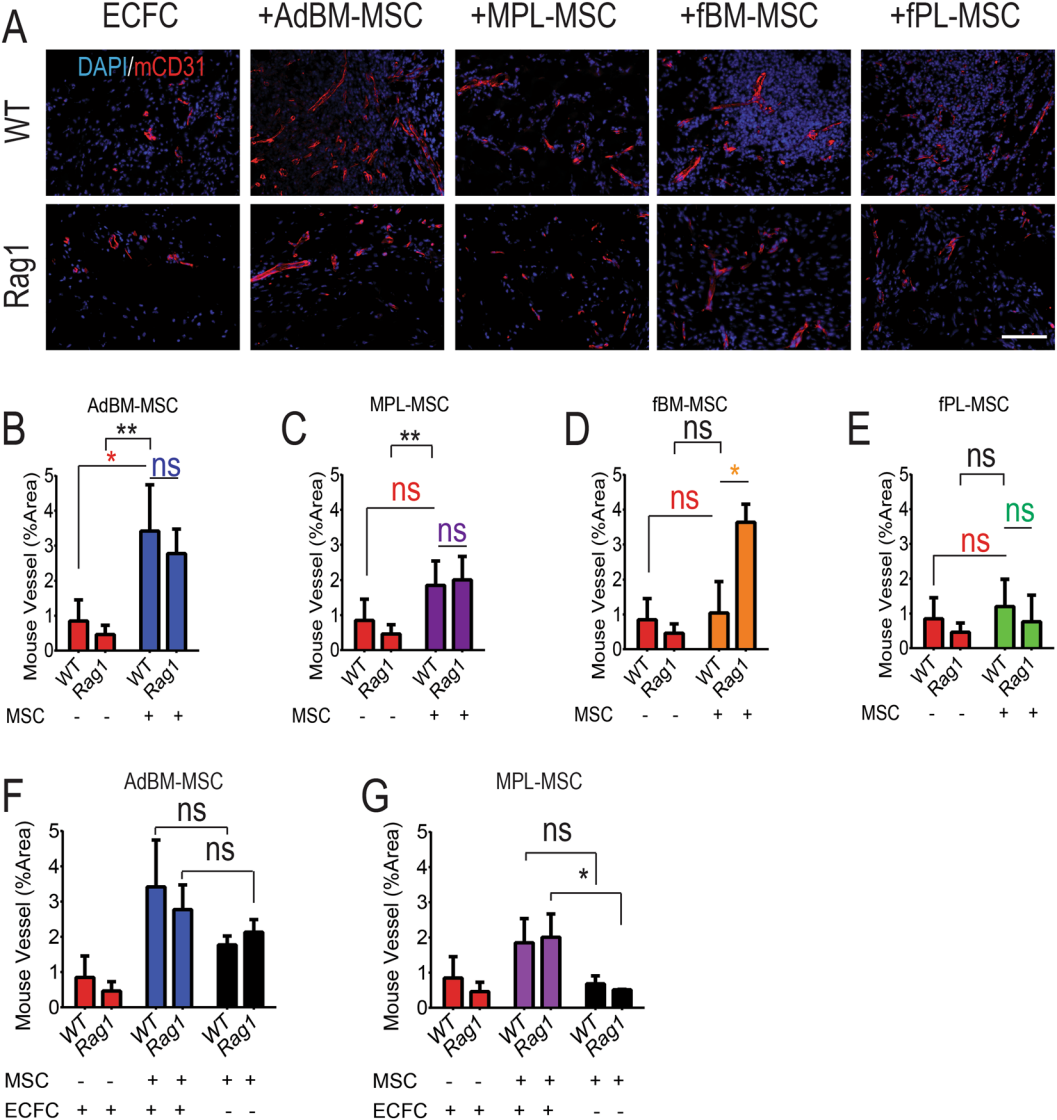
Human stem/progenitor cell paracrine activity supports host neo-angiogenesis regardless of immune status. (**A**) At day 7 after cell engraftment, plugs were harvested and stained with mouse specific anti-CD31 antibody (red). Scale bar: 50 µm. (**B–E**) The surface represented by CD31+ structures was measured as a percentage of the surface of the Matrigel plug on the section. (**B**) Comparison of plugs containing endothelial colony forming cells (ECFC) alone in immunocompetent (C57BL/6: WT) and immunocompromised *rag1* ^*−/−*^ (Rag1) mice. (**C**) ECFC alone versus ECFC co-implantation with AdBM-MSC. (**D**) ECFC alone versus ECFC co-implantation with MPL-MSC. (**E**) ECFC alone versus ECFC co-implantation with fBM-MSC. (**E**) ECFC alone versus ECFC co-implantation with fPL-MSC. (**F**) ECFC alone versus ECFC co-implantation with AdBM-MSC versus AdBM-MSC alone. (**G**) ECFC alone versus ECFC co-implantation with MPL-MSC versus MPL-MSC alone. *P < 0.05, **P < 0.01, ns: non-significant. AdBM-MSC: MSC from adult bone marrow, MPL-MSC: maternal term placental MSC, fBM-MSC: first trimester fetal bone marrow MSC, and fPL-MSC: fetal term placental MSC. Data presented as mean ± SD.

In the ECFC alone group, murine vascularization was enhanced at the transplantation site. However, there was no difference between Rag1 and WT mice (n = 5 mice, Fig. 5A (ECFC group) and Fig. 5B. P = 0.3). MSC co-implantation increased host vascularisation in all groups (Fig. 5A, n ≥ 4 mice for all co-transplantation groups). The AdBM-MSC co-implanted with ECFCs stimulated host vascularization the most (Fig. 5A, +AdBM-MSC). Notwithstanding this, AdBM-MSC co-injection compared to ECFC alone resulted in 6 and 4 fold increase in host vascularization in Rag1 and WT mice respectively (Fig. 5B), while MPL-MSC co-transplantation showed similar mouse vessel formation at the engraftment site, albeit 4 and 2 fold increase compared to ECFC engraftment alone in Rag1 and WT mice respectively (Fig. 5C). Although fBM-MSC co-engraftment had no effect in WT mice, it increased host vascularization for 8 fold in Rag1 (Fig. 5D). Finally fPL-MSC co-injection did not show any support for host vascularization (Fig. 5E). Overall, with the exception of co-implantation with fBM-MSCs, under all other conditions host vascularization was not affected by recipient mice immune status.

Co-implantation with AdBM-MSC and MPL-MSC showed the most impact in mouse vessel formation in Matrigel plug. We therefore evaluated the direct paracrine effect of AdBM-MSC and MPL-MSC when injected alone using Matrigel plug (n = 5 mice). AdBM-MSC delivered alone stimulated mouse vessel formation compared to ECFCs alone in WT and Rag1 mice, however less than ECFC-MSC co-transplantation (all non-significant, Fig. 5F). Moreover, MPL-MSC showed similar mouse vessel formation to ECFCs alone at the engraftment site in both strains (Fig. 5G). However, compared to MPL-MSC alone, co-transplantation of MPL-MSC and ECFCs resulted in a significant increase in mouse vascularization in WT and Rag1 mice, but this was significant only in Rag1 mice (Fig. 5F, P < 0.05).

## Discussion

Restoring blood supply in ischemic tissues is a major goal in many CVD. In this regard, the discovery of endothelial progenitors and their availability in clinically significant quantities has opened the possibility of revascularization of ischemic tissues in atherosclerotic patients using cellular therapy. Transplantation of ECFC with *de novo* vessel formation potential has been central focus of research on therapeutic approaches over the last decade. However, low ECFC engraftment is a major issue that remains to be addressed. Here, we evaluated the influence of the immune system in modulating ECFC cell therapy. We observed significant differences in engraftment and vessel formation capacity of ECFCs between immunocompetent (WT) and immunodeficient (*rag1* ^*−/−*^: Rag1) mice, clearly suggesting an important role of the immune system in impeding ECFC engraftment. Of major importance, this did not affect the paracrine activity of the delivered cells. Finally ECFC engraftment and ECFC derived revascularization in WT mice could be enhanced by co-delivery of MSC isolated from BM but not the placenta. Although essential for translational purposes, the level of ECFC engraftment in WT mice upon co-delivery of MSCs never reached engraftment and vascularisation observed in immunodeficient mice that received both MSCs and ECFCs. This clearly suggests that MSCs act on improving ECFC engraftment and function from additional mechanisms to immunomodulation.

We previously reported that unlike MSC, ECFC were not able to inhibit T cell proliferation in mixed lymphocyte reaction assays^9^. In the current study, ECFC had better engraftment levels in Rag1 mice, suggesting that rejection by the immune system is an important contributor to the low engraftment of ECFC. This was confirmed histologically through fluorescent tracing of engrafting cells but also via bioluminescence to document the viability and metabolic activity of engrafted cells at any given time point. Despite being transplanted in Rag1 mice, the number of ECFCs significantly declined over time suggesting that other factors beyond the immune system are responsible for their progressive loss. Moreover, MSC-ECFC combinations resulted in higher engraftment and vessel formation in Rag1 mice as compared to WT mice in all experiments. This further suggests that MSCs did not abrogate the effects of the immune system. Furthermore the major increase in ECFC engraftment in Rag1 mice upon MSC co-delivery further argues for a non-immune role of MSCs in supporting ECFCs. Notably, our *in vitro* results showed ECFC-induced T cell proliferation was not reduced after MSC co-culture suggesting that the observed improved ECFC engraftment in immunocompetent mice may not be associated with T cell functions. In contrast to our results Souidi *et al*. recently investigated the allogeneic T cell responses against gamma-interferon stimulated ECFC and showed reduced T cell proliferation after co-culturing with MSC as a result of HLA downregulation as well as indolamin-2,3-dioxygenase activation^23^. Although, our results could support Souidi *et al*. findings in regards to improvement of ECFC engraftment level in co-transplantation condition, however, the different outcomes observed in our study may be explained by the use of interferon gamma or the use of human-only cells^23^. Overall, our findings suggest MSCs support ECFC engraftment in WT and Rag1 hosts through non-immune mechanisms such as Notch pathway activation as recently described^24^. Their immunomodulatory activities might be context dependent and cannot fully explain the observed effects.

Our study has therefore limitations in identifying the respective role of immunomodulation versus supportive niche of combined MSCs. However it could be argued that these are intricate mechanisms difficult to separate. The immunomodulatory properties of MSC have been well established^29–32^. Of interest, MSC co-transplantation clinically has improved hematopoietic stem cell engraftment at allogeneic transplantation, in particular for the treatment of severe acute graft-versus-host-disease (GVHD)^33,34^. Allogeneic and autologous MSC co-transplantation also resulted in significant improvement in allogeneic pancreatic islet engraftment in animal models^35–37^. Furthermore, the innate immune system may also be influenced by MSCs and notably NK cells might account for this reduction in ECFC numbers in immunosuppressed mice. Of interest, BM-MSC from adult or fetal origin could alleviate ECFC rejection in WT mice, whereas MSCs of placental origin whether fetal or maternal could not. This may be related to stronger immunomodulatory potential of BM derived MSCs compared to other sources^38–40^. However, MSCs as discussed also displayed an alternate effect that seemed independent of their immunomodulatory role. Indeed for all MSC subtypes co-transplanted with ECFCs, increased ECFC engraftment levels could be observed in immunodeficient mice. This argues for additional and potentially more important mechanisms beyond immunomodulation for the activity of MSCs in promoting ECFC engraftment.

Developmentally, pericytes contribute to stabilizing the vasculature, indeed the similarity between mural cells and MSC might stem from recruitment of MSC in the nascent microvascular wall^41–44^. Moreover, it is widely held that adult MSC may have a perivascular origin^43^. With co-implantation of ECFC with MSC, we showed enhanced vascularization through both paracrine and human vessel formation in accordance with the literature^22^. It is therefore plausible that MSCs improve ECFC engraftment through non-immune mechanisms. We recently dissected the mechanism by which MSCs might improve ECFC survival in stress conditions and improve their vasculogenic potential by providing a stromal niche that activated Notch signalling^24^.

CVD preclinical therapy studies have been reported to rely at least in part on paracrine factors produced by the transplanted cells that induce host-derived neo-vessels^14,45^. Regardless of the level of engraftment with respect to the host immune status, ECFCs induced similar levels of host-derived vascularization of the graft area. In addition, the paracrine effect of cells was much increased on co-implantation. This can be due to the additive effect of MSC derived paracrine activity, as previously reported upon transplantation of cord blood derived ECFC with the conditioned medium of AdBM-MSC^46^. The comparable levels of paracrine activity is another argument for the use of MSC-ECFC combinations in immunocompetent individuals. This is of importance as in the present study, the effects of MSC-ECFC combination on engraftment have been explored here only for a short period. Longer term experiments need to be conducted using a variety of matrixes beyond Matrigel alone. These are likely to reveal that both MSCs and ECFCs will disappear from immunocompetent hosts at later time points. This further enhances the importance of the paracrine activity of delivered cells.

## Conclusion

Application of ECFC in ischemic diseases would be a promising strategy if the engrafted cells were able to survive and establish new vasculature within an allogeneic host. This would allow their preparation as an off the shelf product to be delivered immediately in situations of emergency such as myocardial infarction. We here show that co-transplantation of ECFCs with MSCs seems to be an effective strategy in improving ECFC survival in immunocompetent hosts. Although MSCs might have immunomodulatory roles, our findings suggest that MSC essentially play a role of supportive cells. Moreover, paracrine activities of the delivered cells was not affected by the immune status of the host. Altogether, these findings pave the way for the allogeneic and off the shelf use of ECFCs.

## Experimental plan

### Ethics statement

All human and animal studies were approved by Royal Brisbane and Women’s Hospital (RBWH) and The University of Queensland ethics committees. Human term placentas were collected from the RBWH from healthy mothers during scheduled caesarean-section delivery after written and informed consent. Twin or pathological pregnancies were excluded.

All methods were carried out in accordance with relevant guidelines and regulations by The University of Queensland. All experimental protocols were approved by The University of Queensland ethics committees.

### Preparation of fetal and adult human stem/progenitor cells

Fetal term placental ECFCs^9^, fPL-MSC, MPL-MSC^47^ and fetal first trimester BM-MSC^48^ were isolated in our laboratory as reported previously. AdBM-MSC were purchased from Lonza (PT-2501, obtained from three male donors, 23, 25, and 41 years old). The adipo-, osteo- and chondrogenic differentiation capacity of MSC was confirmed using standard differentiation protocols^49^. To distinguish between ECFC and MSC upon transplantation in co-transplantation experiments, a dual bioluminescent and fluorescent ECFC line was created using dual reporter lentivectors according to manufacturer’s instructions (System Biosciences, CA). High eGFP positive cells were sorted using FACS Aria 11 u (BD Biosciences, CA) machine upon transduction. This allows monitoring of ECFC using luminescence-based *in vivo* imaging and the GFP marker for histological analysis.

### Subcutaneous engraftment of Matrigel containing cells into mice

A Matrigel Plug assay was used to assess ECFC’s *in vivo* engraftment and function. ECFCs were cultured in Endothelial Growth Medium (EGM2, Lonza, USA) and upon harvest 5 × 10^5^ cells were suspended in 150 µl of Matrigel (BD Biosciences, USA) on ice and subcutaneously injected into isoflurane-anesthetized WT or immunodeficient B6. Rag1^*−/−*^ mice (8–12 weeks old), with each mouse receiving four grafts. After 7 days, mice were euthanized using CO2 inhalation and plugs were collected.

In co-transplantation group, ECFCs and MSCs were cultured separately in EGM2 or Dulbecco’s Modified Eagle Medium (DMEM)/10% fetal bovine serum (FBS, both from Gibco) respectively. Then ECFCs alone (5 × 10^5^ cells) or MSC alone (5 × 10^5^ cells) or ECFC-MSC mixture (1 × 10^6^ cells, 1:1 ratio) were suspended in Matrigel and subcutaneously implanted into WT or Rag1 mice. This cells’ ratio was chosen based on a recent study in our group^24^.

### *In vivo* bioluminescence imaging

To confirm the contribution of MSC to ECFC survival, we conducted luciferase-based *in vivo* imaging. On day 1, 3 and 7 post cell injection, mice were intraperitoneally injected with 200 µl (150 mg/kg) D-luciferin substrate (Caliper Life Sciences Inc, MA). After 10 mins images were taken using Xenogen, IVIS Spectrum Bioluminescent Imaging System (Caliper Life Sciences, MA). Then living image software (Caliper Life Sciences Inc, MA) was applied to measure the bioluminescence. Equal-sized regions of interest (ROI) were drawn around individual plug areas and the rate of light emitted was calculated as total flux (photons/sec). Data were plotted against time.

### Immunofluorescence

After 7 days plugs were harvested and fixed in 4% paraformaldehyde (PFA), soaked in 20% sucrose, and then tissues embedded in Optimum Cutting Medium (OCT, Tissue-Tek, CA). 8-μm cryosections were prepared and stained with anti-human Lamin A/C (1:250 dilution, Biolegend, CA) to visualize human cells using a standard immunohistochemistry (IHC) protocol. In brief, slides were incubated for 2 hours with primary antibody at room temperature (RT), and then washed with phosphate buffer saline (PBS)/Tween. After 45 minutes incubation with secondary antibody at RT, slides were mounted in Prolong Gold reagent with DAPI (Invitrogen, CA) and observed under a Zeiss Axio microscope (Carl Zeiss, North Ryde). Quantification of donor (human) derived vessels in the engraftment area was performed by staining for anti-human CD49f (1:100 dilution, BD Biosciences, MA) and CD144 (1:100 dilution, eBioscience, MA) antibodies. To evaluate host (mouse) derived vessels in the implantation site, slides were stained for CD31 antibody (rabbit anti mouse CD31, 1:50 dilution, BD Biosciences, MA).

### T cell proliferation assays

To assess the impact of ECFCs alone and in combination with fPL-MSC on the proliferation of T cells *in vitro* we employed CFSE dilution assay as reported previously^50^. Here, 10-week-old C57BL/6: WT mice were euthanized and spleens were collected. Then, spleens were crushed and using a 40 µm cell strainer and a syringe plunger splenocytes were isolated. Splenocytes were labelled with CFSE *ex vivo* (Invitrogen, 5 µM, 20 min at 37 °C in the dark). After washing, splenocytes were co-cultured in 24-well plates either alone or with ECFC (splenocytes: ECFC; dilution: 100:1 (1.0 × 10^6^: 1.0 × 10^4^)) or combination of ECFC and MSC (splenocytes: ECFC: MSC; dilution: 100:0.5:0.5(1.0 × 10^6^: 0.5 × 10^4^: 0.5 × 10^4^)) and incubated in EGM2. After 48 h, cells were harvested and labelled with Pacific Blue anti-mouse CD3 Antibody (BioLegend) for 20 min on ice and analysed using a flow cytometry machine (Gallios, Beckman Coulter, CA). Then, CD3 positive cells were gated, and the intensity of CFSE was analysed using Kaluza Flow Cytometry Analysis Software (Beckman Coulter).

### Statistical analysis

Analyses were performed using GraphPad Prism v6.04 software. Data are presented as mean ± SD. Groups were compared for parametric variables by student t-tests and one or two-way ANOVA. Mann-Whitney U test was used for comparison of non-parametric variables. A p-value < 0.05 was considered significant.

## Electronic supplementary material

Supplemental Figures.
