# Supplementary material for: Mesenchymal stem/stromal cells enhance engraftment, vasculogenic and pro-angiogenic activities of endothelial colony forming cells in immunocompetent hosts
Source: Sci Rep. 2017 Oct 19;7:13558. doi: 10.1038/s41598-017-13971-3 (PMC5648925; doi:10.1038/s41598-017-13971-3)
Supplement: Supplementary file 1 — Supplemental Figures. [file 41598_2017_13971_MOESM1_ESM.pdf]

Supplemental Information

# *Title:* ***Mesenchymal stem/stromal cells rescue engraftment, vasculogenic and pro-angiogenic activities of endothelial colony forming cells in immunocompetent hosts***

Authors: Abbas Shafiee, Jatin Patel, James S Lee, Dietmar W. Hutmacher, Nicholas M Fisk, Kiarash Khosrotehrani

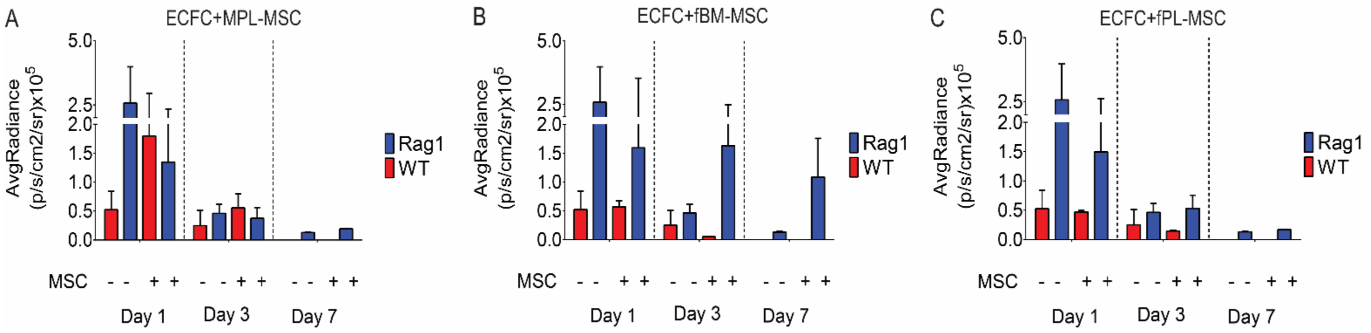


**Supplemental Figure 1:** Co-transplantation with mesenchymal stem cells (MSC) results in increased endothelial colony forming cells (ECFC) survival. A-C) GFP-tagged ECFCs were transplanted in immunocompetent (C57BL/6: WT) and immunocompromised *rag1*$^{-/-}$ (Rag1) mice alone or with A) Maternal mesenchymal stem cells (MSC) from human term placenta (MPL-MSC), B) first trimester fetal bone marrow (fBM-MSC), and C) Fetal MSC from human term placenta (fPL-MSC) and live cell imaging was conducted in days 1, 3, and 7 post engraftment. Data presented as mean ± SD.

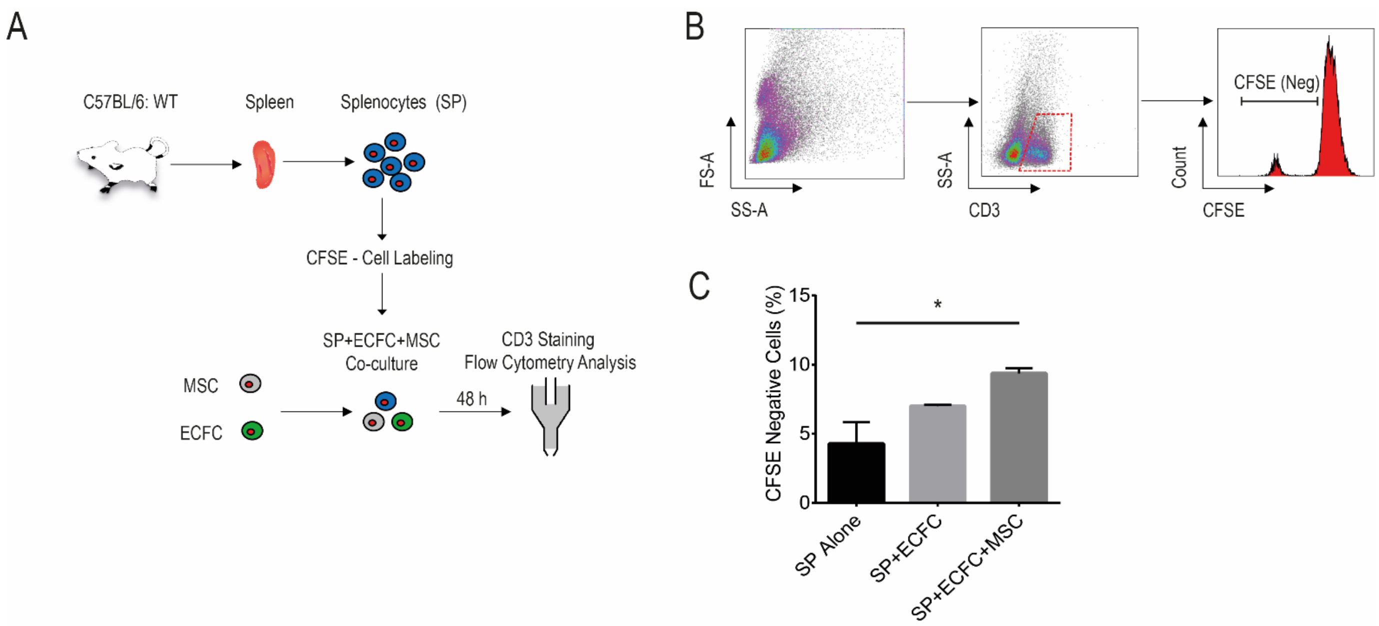


**Supplemental Figure 2:** Mesenchymal stem/ stromal cells (MSC) do not suppress T cell proliferation. A) C57BL/6: WT mice were euthanized and splenocytes (SP) isolated. SP were labelled with the Carboxyfluorescein diacetate succinimidyl ester (CFSE). SP were then co-cultured with endothelial colony forming cells (ECFC) (splenocytes: ECFC; dilution: 100:1) or combination of ECFC and MSCs (splenocytes: ECFC: MSC; dilution: 100:0.5:0.5). After 48 h, SP were harvested and labelled with CD3 antibody and analysed by flow cytometry. B) CD3 positive cells were gated, and the intensity of CFSE was measured. C) Quantification of CFSE negative CD3+ cells after 48 h (n=4, graphs show mean +/- SD).

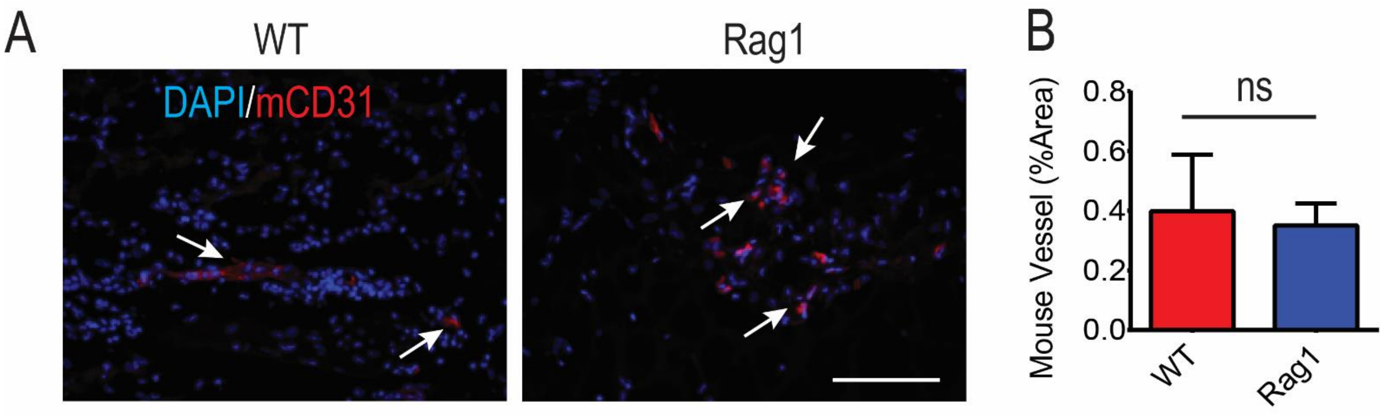


**Supplemental Figure 3:** Mouse vessel formation in Matrigel plugs with no cells implanted. A) To evaluate the impact of Matrigel on host vascularization, Matrigel was subcutaneously injected into mice and at day 7 plugs were harvested and evaluated with mouse specific anti-CD31 antibody. Arrows indicate CD31+ cells. Scale bar: 50µm. B) In the absence of any cell delivered, no difference in host vascularization could be found in immunocompetent (C57BL/6: WT) and immunocompromised *rag1*$^{-/-}$ (Rag1) mice (ns: non-significant, n=5 mice in each strain). Data presented as mean ± SD.
